# Teleological Reasoning in 4-Month-Old Infants: Pupil Dilations and Contextual Constraints

**DOI:** 10.1371/journal.pone.0026487

**Published:** 2011-10-19

**Authors:** Gustaf Gredebäck, Annika Melinder

**Affiliations:** 1 Department of Psychology, Uppsala University, Uppsala, Sweden; 2 Department of Psychology, University of Oslo, Oslo, Norway; University of Bologna, Italy

## Abstract

Four-month-old infants were presented with feeding actions performed in a rational or irrational manner. Infants reacted to the irrational feeding actions by dilating their pupils, but only in the presence of rich contextual constraints. The study demonstrates that teleological processes are online at 4 months of age and illustrates the usefulness of pupil dilations as a measure of social cognitive processes early in infancy.

## Introduction

Humans possess a remarkable range of social cognitive skills that help them understand the goals and intentions of others. Some of these abilities manifest themselves early in development. New-born-infants demonstrate sensitivity to eye contact [1], emotional expressions in faces [2], and goal directed manual actions [3]. Three-month-old infants have been demonstrated to encode action goals [4], evaluate interactions between animated agents in terms of positive or negative [5], and follow others gaze direction [6], [7]. Other social cognitive abilities have, to date, been documented from 6 or 6.5 months of age. These abilities include the tendency to anticipate the goals of others' actions [8] and to detect rational goal directed actions [9], [10], [11]. Rationality is here referred to as taking the most direct and functional path to the goal, given environmental constraints. In the current paper, we focus on the latter of these abilities, more specifically, young infants' tendency to react with surprise when actions are being carried out in an irrational manner.

The first study to demonstrate sensitivity to the rationality of others' actions early in development [11] involved habituation of 6.5-month-old infants to a human walking around a barrier to reach a goal. In a subsequent test phase infants were presented with the same agent walking towards the goal without the barrier. The agent either walked directly to the goal (rational action) or took the old, non-linear, path even though a barrier did not block direct access (irrational action). In response to these events infants looked longer (dishabituated) when observing the irrational detour action relative to the rational straight path action. A similar reaction to irrational detours has been demonstrated at 6.5 months of age when the actions were performed by a robot [11] and a self-propelled box [9]. The flexibility of these processes, operating during observation of humans, robots, and moving boxes, suggest that prior experience with the events being observed is not always required for eliciting dishabituation to irrational agent-goal interactions.

These studies illustrate that 6.5-month-olds are sensitive to abstract principles that operate in the social domain. Infants assume that agents move on rational paths to reach a goal. This assumption is violated when an agent detours from the most direct path. When observing such events infants become surprised, as measured by increased looking times and dishabituation [12]. The notion that infants process the rationality of perceived events is captured by the teleological stance perspective; describing how infants perceive goals and detect irrational or non-functional events without requiring experience with the actual events being observed [13].

Similar principles have also been demonstrated using pupil dilations in both 6 and 12 month old infants. In fact, Gredebäck and Melinder [10] assessed infant's reactions to irrational and rational feeding actions, using corneal reflection eye tracking. In a first experiment infants observed one adult feeding another adult some pieces of banana in an *unconstrained context*, meaning that no barriers informed the observer about rationality. This feeding action could be carried out in a rational manner: the spoon was moved from a plate to the recipient's mouth without unnecessary detours; or an irrational manner: the food was directed toward the back of the recipients hand prior to being placed in the mouth (i.e., the recipient leaned forward and ate from the back of her hand). In response to seeing the irrational detour (plate-hand-mouth) infants dilated their pupil. This dilation is seen as a marker of enhanced focused attention caused by enhanced information processing load and/or arousal [14], [15], [16].

In a second experiment the source of this pupil dilation was further explored in a *constrained context*. In this context the feeding action was always directed towards the back of the hand and the location of a barrier defined the degree of rationality. In one condition the barrier was placed outside the action space of the two agents, maintaining the irrational aspect of the feeding action. In another condition the barrier blocked the direct path to the mouth while allowing direct access *only* to the recipient's hand. In this latter case the feeding action, directed towards the back of the recipient's hand, became *more* rational, given environmental constraints. Results were that 6-month-old infants dilated their pupil more when observing the irrational feeding than during the more rational feeding. The fact that many of the infants in this study had not been fed with solid foods (e.g., with a spoon) on a regular basis suggests that fundamental components of teleological processes might be activated independently of experience with the specific actions being observed [10].

The current study asks, for the first time, to what extent teleological processes are operational in infants below 6 months of age. In order to answer this question 4-month-old infants were presented with 4 different conditions, equaling the rational and irrational conditions of the unconstrained and constrained contexts used by Gredebäck and Melinder [10]. Based on the findings above, we predict that infants will dilate their pupil more during observation of irrational than rational conditions in both constrained and unconstrained contexts.

## Methods

### Participants

Twelve 4-month-old infants (6 girls), mean age 134 days, range 122–154 days, participated in the final sample. One additional boy (122 days) was excluded due to lack of gaze data. Only two of the infants had ever been fed with a spoon (once/day for 6 weeks and twice/day for 2 weeks), none were eating on their own. Most infants had, however, observed others eat on a regular basis (on average 1.8 times/day, range 0 to 3 times/day, for the last 2.7 months, range 0 to 4 months).

### Stimuli and design

All infants were presented with 4 different conditions in which one actor (feeder) scoops up a piece of banana from a plate with a spoon and waits while a second actor (recipient) simultaneously opens her mouth and moves her hand sideways on a table. Following the completion of this action the feeder says, “here it comes” and subsequently moves the spoon across a table to the recipient (duration of reaching actions range from 760 to 1040 ms) who eats the piece of banana and return to her original position (arm is moved back and the mouth is closed). The feeding action is repeated three times in succession. Each movie (including three feeding actions) lasted ∼50 seconds.

Two of the conditions feature an unconstrained context without barriers or other obstacles that restrict the action space of the feeder. During rational feeding within the unconstrained context the feeder place the piece of banana inside the recipient's mouth (*rational and unconstrained condition*). During irrational feeding within the unconstrained context the spoon is directed towards the back of the recipient's hand. In the latter case the recipient leans forward and eats the banana off the back of her hand (to ensure an equal goal state in both conditions) once the feeder's hand has been retracted and is resting on the table (*irrational and unconstrained condition*).

**Figure 1:**
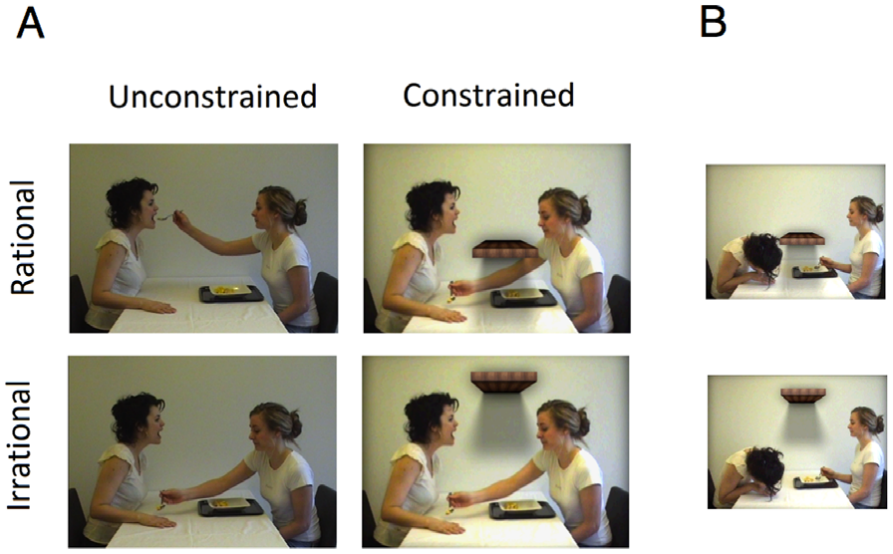
Snapshots of the feeding action; bringing food to the recipients hand/mouth. (A). Snapshots of the recipient leaning forward to eat off the back of her hand (B) in the irrational and constrained condition (upper) and the rational and constrained condition (lower).

Two other conditions feature a constrained context in which a barrier is superimposed onto the irrational and unconstrained condition described above. Accordingly, the same feeding to the back of the hand action is displayed, but this time with a barrier extending from the wall behind the actors. During irrational feeding the barrier is placed outside the actors reaching space (*irrational and constrained condition*). During rational feeding the barrier is placed in a manner that restricts the feeder's access to the recipient's mouth (*rational and constrained condition*). Snapshots of the four conditions are presented in Figure 1.

### Procedure

Upon entering the lab parents received a verbal and written explanation of the study, several verbal questions about their infant's prior experience being fed and observing others eat (see Participants above), after which parents signed a consent form in accordance with the 1964 declaration of Helsinki. Following a 5-point calibration procedure infants were presented with 6 movies from one condition followed by a short break and 6 movies from another condition. For example, if an infant was initially presented with 6 movies (each including 3 feeding actions) from the *rational and unconstrained condition* the same infant might then be presented with 6 movies from the *irrational and constrained* condition. On the next day the same infants are presented with the remaining conditions. In the example above this would include 6 movies of the *irrational and unconstrained condition* and 6 movies from the *rational and constrained condition*. All infants were thus presented with 18 feeding action (3 feeding actions ×6 movies) from each of the four conditions, in a counterbalanced order over the two sessions (a total of 72 feeding actions; 18 feeding action ×4 conditions). Participating families received a gift voucher (∼12€) as compensation.

### Data reduction

Two time windows were defined anchored around the initiation of the feeding action (extending the arm with the spoon towards the recipient). The baseline block covered the 4 seconds prior to this event and the test block covered the following 4 seconds, including the reaching action, the contact between the spoon and the recipient, ending approximately when the hand retracts from the hand/mouth (see Figure 1).

One participant's average pupil diameter was substantially smaller than remaining participants (>3 z-scores); he was removed from further analysis. For remaining 12 infants data was included for 98%, 87.5%, 89.6%, 92.7% of trials for irrational and unconstrained, rational and unconstrained, irrational and constrained, and rational and constrained conditions, respectively.

Changes in pupil diameter were estimated by subtracting the average diameter in the test block from the average diameter in the baseline block, aggregated over all feeding events included for each stimulus. Prior to statistical analysis 2 aggregated data points were replaced (>2.5 z-scores) with group means. Preliminary analysis did not find any effects of presentation order; data was, as such, aggregated over trials. Two paired sample t-tests were used. One examined the extent to which infants dilated their pupil more during the irrational and unconstrained conditions than the rational and unconstrained conditions. Another examined the extent to which pupil dilations were more pronounced during the irrational and constrained condition relative to the rational and constrained condition. In addition, single-sample t-tests against zero were used to investigate if the pupil dilated significantly from baseline.

## Results

**Figure 2:**
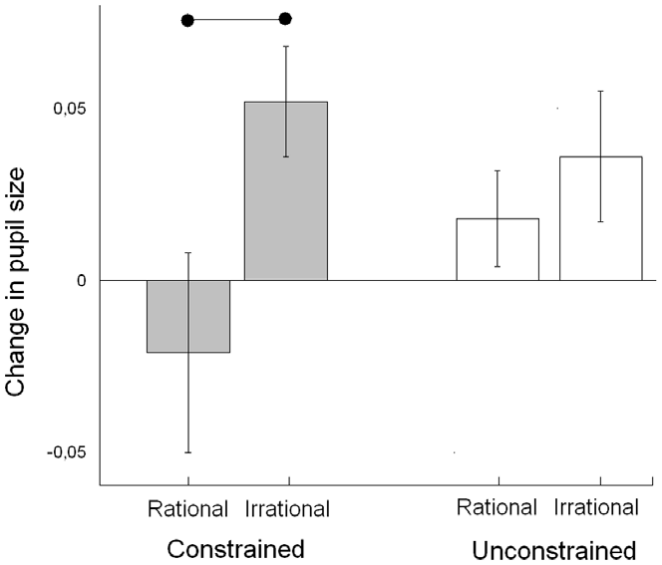
Average change in pupil size. Measured from baseline to test block, separate for the rational and irrational conditions of the unconstrained and constrained contexts. Error bars represent SE and the horizontal line represent conditions that significantly differ from each other.

Ten out of 12 infants dilated their pupil from baseline to test block during observation of the irrational and constrained condition (sign-test *p*<.05). Only half of the infants dilated their pupil during observation of the rational and constrained condition (pupil dilation in 6/12 participants, n.s.). Comparing the rational and irrational conditions of the constrained condition demonstrated more pupil dilation during observation of the irrational and constrained than the rational and constrained condition, *t*(11) = 2.48, *p* = .03, *d* = .89, see Figure 2. Pupil dilations differed from zero in the irrational and constrained condition, t(11) = 3.35, p<.01, but not during the rational and constrained condition.

At the same time infants did not differentiate the rational and unconstrained condition from the irrational and unconstrained condition, *t*(11) = 0.66, *p* = .52, *d* = .32. Pupil dilations did not differ from zero for either the rational and unconstrained or the irrational and unconstrained conditions.

## Discussion

The present study is the first to demonstrate sensitivity to violations of rationality at 4 months of age. Infants dilated their pupil when observing one person feeding another by placing food on the back of her hand, but only when a barrier was present that did not restrict the feeder's access to the recipient's mouth. These findings support the claim that teleological principles are online by 4 months of age. The early emergence of this ability demonstrates that young infants are equipped with a large cognitive toolbox that helps them interpret real world events.

### Pupil dilation

Few studies have relied on pupil dilations to investigate the developing mind, and only two studies [10], [17] have applied this measure to infants' social cognitive abilities. There have, however, been some recent reports that relate pupil dilations to face processing in children with autism [18] and object representations in infancy [19]. We believe this methodology provides an excellent supplement to conventional looking time measures, the main benefits being that reactions are assessed during observation of single events in real time, not following the completion of a series of events. This allows numerous data points to be collected in a short time (each block [4 seconds] includes 200 samples ×3 feeding actions ×6 movies, equals up to 3600 data points/block and stimuli) allowing a substantial reduction of noise that facilitates detection of early developing mental processes.

Currently, our understanding of the relation between pupil dilation and underlying processes are far from complete. However, a recent review suggests that changes in arousal or information processing load lead to alterations in focused attention and time locked pupil dilations; the connection between altered attention and pupil diameter mediated by the norepinephrine system and the locus coreuleus [15].

Before any claims are made about the specific cognitive processes that might lead to altered states of attention and pupil dilations it is paramount to control for differences in luminance. In general, increasing the luminance contracts the pupil whereas decreasing the luminance dilates the pupil [20]. The goal area (back of the hand) had a luminance of ∼200 fL in the irrational and unconstrained condition, the irrational and constrained condition, and the rational and constrained condition. The goal area in the rational and unconstrained condition was less bright (mouth ∼150 fL hair ∼20 fL). A luminance account would, as such, predict larger dilation to the rational and unconstrained condition than the other conditions. As demonstrated in Figure 2 this prediction does not agree with the results. Of the three conditions where pupil dilations were observed (on average) the rational and unconstrained condition demonstrated the smallest dilation of the pupil.

An alternative luminance account might be that the relative size of the pupil is influenced by the luminance at the middle of the screen, where the hand and spoon moves across the table towards the goal area. Here the biggest difference exists between the two constrained conditions, due to the different locations of the barrier. However, as above, the condition in which the shadow of the barrier makes the path to the goal darker is the condition with the smallest pupil diameter. In fact, the average response to the rational and constrained condition was a contraction of the pupil.

Given that luminance most likely cannot account for the current pattern of pupil dilations we argue that pupil dilations, in the current study, is driven by alterations in focused attention that might be influenced by several factors including enhanced cognitive load, enhanced emotional processing, and reactions related to violations of rationality principles. Future research is essential to map out the relationship between changes in pupil diameter and the cognitive process that give rise to this reaction.

### Teleological processes and the four-step action segmentation model

The current findings suggest that infants already at 4 months of age are able to interpret other people's actions as rational or irrational. These findings bare similarities and differences to prior studies using the same stimulus material with older infants [10].

Conceptually similar for both studies (the current findings and [10]) is that infants do not require a learning phase in which agents act rational prior to being tested on irrational events (as is common to prior studies of teleological reasoning using habituation techniques, for example [9], [11]). In Gredebäck and Melinder [10] infants were only presented with a single condition and reactions to seeing irrational social interactions were measured across infants. In the current study infants were presented with all conditions in a within-subject design, however, in a counterbalanced presentation order without significant learning effects. Together these studies suggest that infants bring expectations about how agents should act towards each other to the lab. These expectations are not dependent on a specific action or social interaction but rather adaptable to a wide range of social events, even to events that infants have not encountered before.

If infants do not base their reactions on prior exposure to irrational feeding events, what makes them react to violations of rationality in this context? We suggest a *four-step action segmentation model* that allows 4-month-old infants to react to irrational social actions or social interactions without direct experience with the actions being observed. We suggest that infants are able to (1) segment perceived social interactions into action units, such as a reaching action, leaning forward, and eating. (2) At the same time we suggest that infants are able to detect the overall action goal; that is to feed the recipient. (3) Encoding individual action units as they occur might allow infants to evaluate the efficiency of each action unit against the overarching action goal. In the case of functional action sequences (for example the rational conditions of the current experiment) each action unit brings the overall goal closer to completion. In the case of non-functional action sequences some action units (in this case bringing the food to the back of the receiver's hand) bring the overarching goal further away from completion (here defined as a spurious action unit). (4) Detection of spurious action units violate assumptions that action sequences are carried out in the most functional manner possible given environmental constraints (as described by the teleological stance [9]), causing a surprise reaction, enhanced focused attention, a surge of norepinephrine and dilated pupils [15].

Though speculative, there is evidence suggesting that infants are able to segment action sequences into action units already at 6 months of age [21], for more studies with 10–11 month olds see [22], [23]. To our knowledge no study has investigated action segmentation in 4 months olds, opening up for the possibility that similar processes are available to infants participating in the current study.

At the same time we know that infants at both 3 and 5 months of age are able to encode individual action goals [24], [25], [26]. It is also clear that older infants are able to incorporate the overall goal of an observed action sequence into their own behavior. For example, 14-month-old infants' ability to anticipate the goal of a reaching action is dependent on how the goal object is later used [27]. Whether 4-month-olds are able encode the overarching goal of an action sequence is less clear.

In order to validate the suggested *four-step action segmentation model* it is required that 4-month-old infants are able to evaluate the efficiency of individual action units against the overarching goal of the action sequence. Future research is required to assess the degree to which 4-month-old infants are able to perform the processes here suggested.

### The origins of teleological processes

Regardless of the validity of the proposed model it is clear that 4-month-old infants are sensitive to the rationality of the perceived social interaction. It is currently unclear if this sensitivity to irrational events is experience independent in accordance with core knowledge theory proposed by Spelke [28] or rooted in early experience with rational action sequences and rational social interactions in general. In accordance with the latter suggestion, it is possible that a substantial exposure to a large variety of functional combinations of action units have resulted in a generalized expectation that actions are carried out in the most efficient manner possible, given environmental constraints [29]. In short, it is unclear at this stage if the demonstrated early sensitivity to irrational social interactions is innate or based on early experience with rational actions.

### Development of teleological processes

Infants undergo substantial development between 4 and 6 months of age. Six-month-olds are able to react to irrational actions without the presence of barriers (Experiment 1, [10]) whereas 4-month-olds (in the current study) are only sensitive to irrational action in the context of a barrier. One interpretation of this finding might be that the barriers helped conceptualize the contextual constraints, even when observing unconstrained feeding to the back of the hand. Phrasing this suggestion in terms of the four-step action segmentation model postulated above it is possible that the barrier facilitates the efficiency evaluation of individual action units with respect to the overarching action goal. An alternative and perhaps complementary assumption is that infants simply perceived the back of the hand as a feasible goal in the unconstrained condition (which is not the case for 6- or 12 month-old infants according to prior findings [10]). If this was the case infants should not dilate their pupil. It is, as such, an open question as to what extent the null effect during the unconstrained context represents a rational assessment based on perceived immediate goals or an inability to evaluate the efficiency of the action sequence in the absence of clear visual markers, in this case the barrier. A comparison with the findings of Gredebäck and Melinder [10] demonstrates that 6-month-old infants are able to apply teleological processes to more diverse contexts (involving both the constrained and unconstrained context) than 4 month-olds.

### Summary

For now, we argue that the pupil dilations elicited during observation of irrational social interactions in the current study represent the earliest markers of teleological processes present in the literature and that teleological processes, in turn, represent an early emerging abstract social cognitive mechanism devoted to comprehending the goals and intentions of others.
